# New population-specific cephalic index standards for Malaysian subadults: prevalence, growth patterns, and clinical implications from a CT imaging study

**DOI:** 10.1016/j.clinsp.2025.100855

**Published:** 2025-12-19

**Authors:** Sharifah Nabilah Syed Mohd Hamdan, Rabi’ah Al-Adawiyah Rahmat, Selva Malar Munusamy, Norliza Ibrahim

**Affiliations:** a Department of Oral and Craniofacial Sciences, Faculty of Dentistry, Universiti Malaya, Kuala Lumpur, Malaysia; b Department of Oral and Maxillofacial Clinical Sciences, Faculty of Dentistry, Universiti Malaya, Kuala Lumpur, Malaysia; c Department of Restorative Dentistry, Faculty of Dentistry, Universiti Malaya, Kuala Lumpur, Malaysia

**Keywords:** Cephalic index, Malaysian subadults, Deformational brachycephaly, Cranial deformity, Computed tomography

## Abstract

**Objective:**

Population-specific Cephalic Index (CI) classifications are lacking for Malaysian subadults. This study aimed to develop a new CI classification and determine the prevalence of deformational brachycephaly using Computed Tomography (CT) images.

**Methods:**

A total of 520 CT images from subadults aged 0- to 20-years were included in the study. Two cranial measurements were obtained: the maximum Cephalic Length (CL) and the maximum Cephalic Width (CW), derived from axial CT images. The CI was subsequently calculated using the formula: CW/CL × 100. In addition, a simple linear regression analysis was performed to evaluate the relationship between CI and age.

**Results:**

The modified CI ranges for the Malaysian subadult population were defined as follows: dolichocephalic ≤ 78.8, mesocephalic 78.9–89.0, brachycephalic 89.1–94.0, and hyperbrachycephalic ≥ 94.1. The predictive equation derived was y = 87.08–0.47x +0.01×^2^ (y = CI and x = Age in years). A significant negative correlation was found between age and CI (*r* = -0.101, p < 0.001), with the index decreasing by approximately 0.026 per year. This indicates a natural trend towards a less brachycephalic head shape with maturation.

**Conclusion:**

This new, population-specific classification provides an essential reference tool for clinicians and researchers to accurately detect, manage, and treat cranial deformities in Malaysian subadults, with significant applications in pediatrics, anthropology, and forensic medicine.

## Highlights

• Establishes the first cephalic index standards for multi-ethnic Malaysian subadults.
• Reports a negative correlation between age and CI, with a decline of 0.026 yearly.
• Shows higher CI values in Malaysians than in Caucasians and other Asian groups.
• Provides a validated tool (ICC > 0.93) to track cranial growth and deformity severity.

## Introduction

Deformational Brachycephaly (DB) is a prevalent condition in pediatric neurology and orthopaedics. It involves the abnormal flattening of the back of an infant's head. The incidence of this condition has increased since the 1990s, particularly following the introduction of the ‘Back to Sleep’ campaign, which was designed to reduce Sudden Infant Death Syndrome (SIDS)^1^. It has since become a significant concern for both parents and healthcare providers^2^. DB is marked by a symmetrical flattening of the occiput, typically resulting from external pressure on the skull. This pressure causes compensatory widening of the parietal region, leading to a disproportionately short and broad head shape^3^. Beyond affecting cranial aesthetics, DB also raises concerns about its potential impacts on neurological development and psychosocial well-being.

Cephalic Index (CI) was invented and first used in Europe to classify the shape of the skull^4^. It also aims to evaluate the efficacy of interventions aimed at correcting cranial deformations in children, such as the utilization of helmets to address brachycephaly^5^. The concept of CI can be defined as the proportion of a skull’s maximum width to its longest length, multiplied by 100^6^. The CI value to classify brachycephaly has been reported to vary from 81 % to 95 %^5^^,^^7^^,^^8^. In the Asian population, brachycephalic head shapes are more prevalent, partly due to the customary supine sleeping position, resulting in an extreme cephalic index exceeding 100 %^7^, ^8^, ^9^, ^10^. The definition of brachycephaly based on CI lacks standardization^8^. This highlights the need for Malaysia to develop its own CI classification for subadults.

Initially, CI was measured using a calliper or plain radiographs. However, this method has several limitations, including distortions, lack of perspective, structural superimposition, magnification, rotational, and head positioning errors^11^. Hence, Computed Tomography (CT) scan is the preferred tool over other imaging due to high-quality visualization of the underlying bony architecture^12^. In addition, Three-Dimensional (3D) CT scans aid surgeons in identifying bony abnormalities and permit a more detailed analysis of asymmetric structures, segmental movements using 3D digital operations^13^. Early diagnosis of DB is important for assessing prognosis and devising treatment strategies. Delay in diagnosis can potentially result in significant complications, including neurodevelopmental impairment and intracranial hypertension^14^. Therefore, this study aimed to develop a new CI classification and determine the prevalence of deformational brachycephaly using CT images.

## Materials and methods

### Ethical approval

Study approval was obtained from University Malaya Medical Centre’s Ethics Committee (approval number 202147‒10039). The study was conducted adhering to the principles laid out in the Declaration of Helsinki. Written informed consent was not required, as this requirement was waived by the Ethics Committee due to the retrospective nature of the study. The study was designed, conducted, and reported in accordance with the Strengthening the Reporting of Observational Studies in Epidemiology (STROBE) guidelines.

### Study population

This was a retrospective study consisting of Computed Tomography (CT) images of 520 subadults (278 male and 242 female), comprising 220 Malay, 145 Chinese, and 155 Indian individuals, aged ≤ 20-years. The distribution of sex categorised by age groups is presented in Table 1. The images were obtained from the Radiology Department of University Malaya Medical Centre. Inclusion criteria were brain CT scans performed for suspected head trauma with normal findings, in individuals aged ≤ 20-years and of Malay, Chinese, or Indian ethnicity. Exclusion criteria were CT images of individuals due to a history of trauma, such as head injuries, concussion, hematoma, and skull fracture. Additionally, CT images associated with surgical history, deformities, or other pathological conditions, mental deficiency, and those featuring artifacts or poor resolution were excluded. Subjects were categorized into six age groups: 0‒2 years, 3‒6 years, 7‒9 years, 10‒12 years, 13‒15 years, and 16‒20 years (Table 1).

**Table 1:** Distribution of sex categorized by age groups.

Age groups (years)	0–2	3–6	7–9	10–12	13–15	16–20	Total
Male	60	43	35	30	41	69	278
Female	51	36	22	30	38	65	242
Total	111	79	57	60	79	134	520

### Procedure

High-resolution CT images were acquired using the Philips Ingenuity 128 CT Scanner (Philips Medical System, Cleveland Inc, United States). Scanning parameters included a tube voltage of 120 kV, tube current of 100‒400 mAs, exposure time of 0.4‒0.6 s, and a voxel size of 0.625 × 0.625 × 1 mm. Image reconstruction was performed using a convolution kernel ranging from H40s to H60s. All images were stored in Digital Imaging and Communications in Medicine (DICOM) format. The CT datasets were imported into Mimics software, version 21.0 (Materialise, Belgium).

**Fig. 1:**
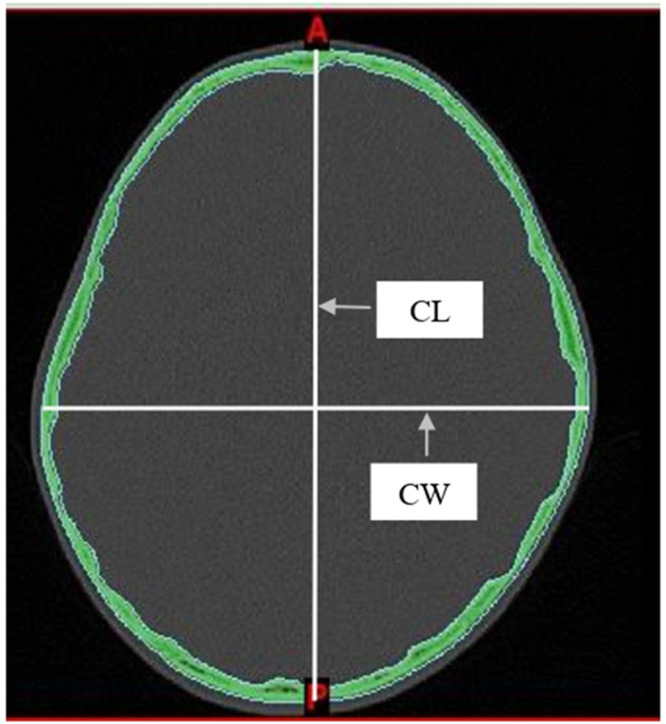
Measurements from axial CT scan. CL, Cephalic Length, CW, Cephalic Width. Cephalic index = CW/CL × 100.

Two measurements were taken from the skull: the maximum Cephalic Length (CL) and the maximum Cephalic Width (CW). These were measured directly from axial CT images using the linear measurement tools in Mimics software. Measurements were taken on a plane parallel to the Frankfurt horizontal (auriculo-orbital) plane. Following the methods described by Waitzman et al^15^, all scans were assessed using established bony landmarks selected to evaluate the cranial vault. The cephalic index was then determined using the equation as follows: cephalic width/cephalic length × 100 (Fig. 1).

According to the classification method proposed by Koizumi et al^7^, CI was classified by employing the data taken, and the CI mean was defined as ±1 Standard Deviation (SD) for mesocephaly, ≤ −1 SD for dolichocephaly, +1 to 2 SD for brachycephaly, and ≥ 2 SD for hyperbrachycephaly. The severity of cranial shape abnormalities was assessed using the percentile distribution method^15^. Dolichocephaly was classified as mild (10th to 25th percentiles), moderate (3rd to 10th percentiles), and severe (below the 3rd percentile). Brachycephaly was classified as mild (75th to 90th percentiles), moderate (90th to 97th percentiles), and severe (above the 97th percentile) (Fig. 1).

### Statistical analysis

SPSS version 25.0 (IBM SPSS, Armonk, NY, USA) was used to conduct all data operations and statistical analyses. Statistical significance value was accepted at *p* < 0.05. The consistency of the investigated variables’ empirical distribution with the normal distribution was evaluated by the Shapiro-Wilk test. Variance homogeneity was assessed using the Levene test. Analysis of variance (ANOVA) was performed to assess the dissimilarities between the age groups. A scatterplot was generated and analyzed using linear regression to assess the correlation between age and CI.

### Observer error assessment

The Intraclass Correlation Coefficient (ICC) was used to assess the reliability of measurements by checking consistency between different raters (inter-rater reliability) or repeated measurements by the same rater (intra-rater reliability). ICC values range from 0 to 1, with higher values indicating higher reliability; scores above 0.75 suggest strong agreement, while those below 0.5 indicate poor reliability. In this study, 70 randomly selected cranial CT datasets from various age groups, sexes, and ethnicities were re-measured by the main investigator with a minimum interval of three weeks to assess intra-rater reliability. Additionally, 40 randomly selected datasets were assessed by an experienced oral and maxillofacial radiologist with over seven years of experience to evaluate inter-rater reliability.

## Results

The Shapiro-Wilk test showed that the p-values for CL and CW were greater than 0.05, suggesting that the data did not significantly deviate from normality. Additionally, Levene’s test for homogeneity of variances showed non-significant results (*p* > 0.05), confirming that the variance across age groups was homogeneous. The ANOVA results showed no statistically significant differences between the age groups (*p* > 0.05). High ICC values were recorded for CI measurements (CL and CW), indicating strong reliability. For intra-rater reliability, ICC values exceeded 0.95. Similarly, inter-rater reliability showed ICC values above 0.93, reflecting a high level of agreement between different observers.

Table 2 shows the mean CI values ± standard deviation across all age groups. The overall mean CI was 84.04 ± 4.71, with females (84.13 ± 4.72) exhibiting a slightly higher CI than males (83.91 ± 4.63). Across age groups, the CI showed a decreasing trend with age, from 86.31 ± 5.37 in the 0–2 years group to 82.03 ± 4.54 in the 16–20 years group. Statistical analysis indicated no significant difference in cephalic index between males and females across all age groups (*p* > 0.05). However, an overall significant difference was observed (*F* = 12.775, *p* < 0.001), suggesting variability in CI measurements among different age categories.

**Table 2:** Descriptive statistics of mean ± standard deviation (minimum‒maximum) values of cephalic index between sexes according to age group category.

Age groups (years)	Cephalic index			*p*-value (male vs. female)	Test statistic
	Total	Male	Female		
0–2	86.31 ± 5.37 (75.2‒01.7)	86.52 ± 5.22 (75.9–96.9)	86.06 ± 5.59 (75.2–101.7)	0.835	
3–6	84.99 ± 4.71 (72.3–96.8)	85.93 ± 4.48 (75.7–93.6)	83.87 ± 4.79 (72.3–96.8)	0.763	
7–9	84.22 ± 4.76 (72.9–92.6)	84.50 ± 4.86 (72.9–92.6)	83.78 ± 4.67 (75.0–92.1)	0.984	
10–12	84.44 ± 4.18 (75.6–93.3)	83.72 ± 3.92 (75.6–93.3)	85.15 ± 4.37 (76.2–92.2)	0.389	*F* = 12.775
					*p* < 0.001
13–15	82.27 ± 4.70 (72.0–92.3)	81.89 ± 4.64 (72.0–92.3)	82.67 ± 4.79 (74.9–91.2)	0.962	
16–20	82.03 ± 4.54 (71.0–93.8)	80.87 ± 4.66 (71.0–91.5)	83.25 ± 4.10 (74.5–93.8)	0.962	
Total	84.04 ± 4.71	83.91 ± 4.63	84.13 ± 4.72	0.815	

Table 3 presents the classification of cranial shape based on CI compared across different studies, including Malaysia, Cohen, Nam, and Koizumi. The modified CI ranges for the Malaysian subadult population were as follows: dolichocephaly (≤ 78.8), mesocephaly (78.9–89.0), brachycephaly (89.1–94.0), and hyperbrachycephaly (≥ 94.1). In comparison, Cohen's classification had a lower dolichocephaly threshold (≤ 75.9) and a narrower brachycephaly range (81.0–85.4), with hyperbrachycephaly defined as ≥ 85.5. Nam's classification set a slightly higher dolichocephaly cutoff (≤ 80.1), with mesocephaly (80.2–93.4), brachycephaly (93.5–100.0), and hyperbrachycephaly (≥ 100.1). Koizumi's classification showed a wide cephalic index range: mesocephaly (79.2–93.8), brachycephaly (93.9–101.1), and hyperbrachycephaly (≥ 101.2).

**Table 3:** Comparison between the proposed cephalic index classification and other existing cephalic index classifications.

Cranial shape	Malaysian	Cohen's	Nam's	Koizumi's
Dolichocephalic	≤ 78.8	≤ 75.9	≤ 80.1	≤ 79.1
Mesocephalic	78.9‒89.0	76.0‒80.9	80.2‒93.4	79.2‒93.8
Brachycephalic	89.1‒94.0	81.0‒85.4	93.5‒100.0	93.9‒101.1
Hyperbrachycephalic	≥ 94.1	≥ 85.5	≥ 100.1	≥ 101.2

Table 4 presents the method for evaluating cranial deformity severity in Malaysian subadults using percentile ranges. For dolichocephaly, mild cases fall between 77.0 and 80.7, moderate cases between 75.1 and 76.9, and severe cases at 75.0 or below. For brachycephaly, mild cases range from 87.3 to 90.3, moderate cases from 90.4 to 93.4, and severe cases at 93.5 or above.

**Table 4:** Classification of cranial deformity severity in Malaysian subadults using percentile ranges.

Classification	Cephalic index	
	Dolichocephaly	Brachycephaly
Mild	77.0‒80.7	87.3‒90.3
Moderate	75.1‒76.9	90.4‒93.4
Severe	< 75.0	> 93.5

**Fig. 2:**
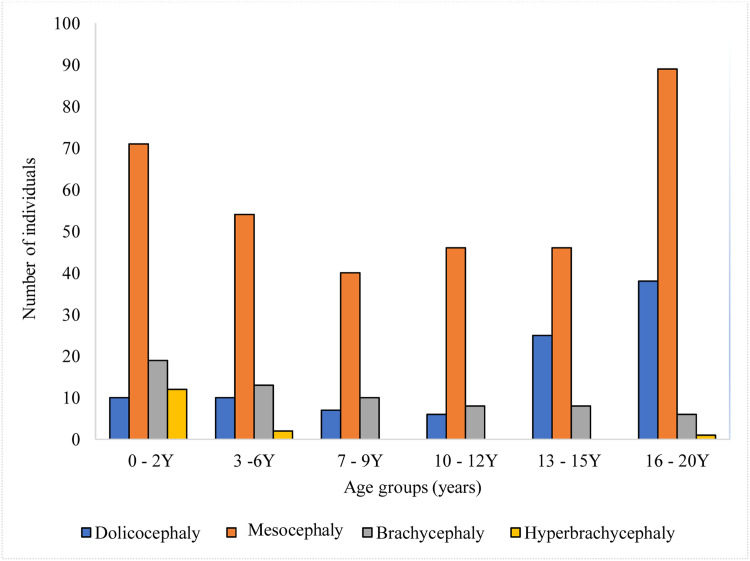
The cranial shapes of Malaysian subadults by age groups.

Fig. 2 illustrates cranial shapes across different age groups. The predominant head shape observed in Malaysian subadults was mesocephaly, consistently dominant from early childhood (0–2 years) through late adolescence (16–20 years), with the highest frequency in the 16–20 years age group. Dolichocephaly had a relatively lower prevalence in younger age groups but increased significantly during adolescence (13–15 and 16–20 years age groups). Brachycephaly was present across all age groups but remained less common than mesocephaly. Hyperbrachycephaly was the least common cranial shape across all age groups.

**Fig. 3:**
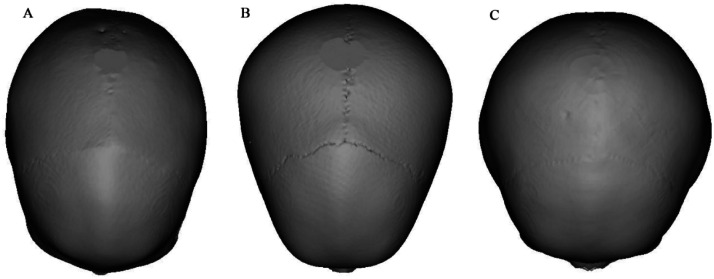
Three-dimensional reconstructed images of the skull showing various shapes and structures (A) Dolichocephaly (elongated skull with a lower CI), (B) Mesocephaly (balanced skull shape with a moderate CI), (C) Brachycephaly (wider, shorter skull with a higher CI).

**Fig. 4:**
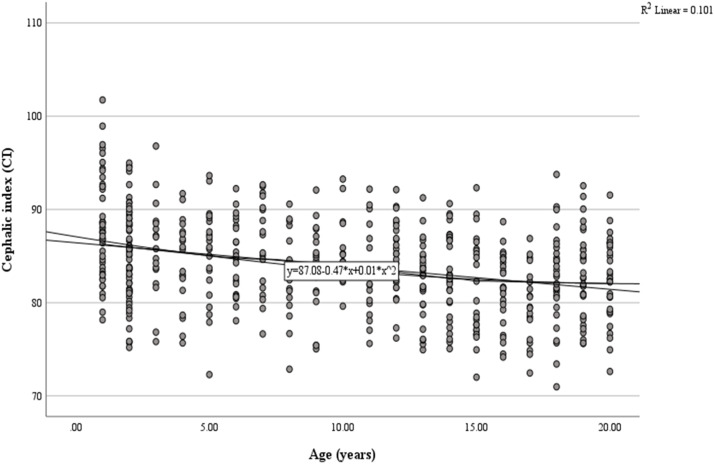
Scatterplot of simple linear regression for CI by age (years).

Fig. 3 shows 3D skull reconstructions of different cranial shapes: dolichocephaly (A), mesocephaly (B), and brachycephaly (C). Dolichocephalic skulls are elongated with a lower CI, while mesocephalic skulls have a balanced shape with a moderate CI. Brachycephalic skulls are wider and shorter, with a higher CI. Fig. 4 presents a scatterplot illustrating the negative and linear relationship between age and CI. Statistical analysis revealed a significant correlation between age and CI (*r* = 0.101, *p* < 0.001). The equation for predicting CI: *y* = 87.08–0.47x+0.01×^2^ (y: CI, x: Age in years). Based on the regression model, the CI was observed to decrease by 0.026 each year (Tables 2, 3, and 4, Figs. 2, 3, and 4).

## Discussion and conclusion

Cephalic Index (CI) is a valuable measurement for determining the shape of a skull. It plays a crucial role when comparing individuals of different ages, sexes, and population affinities in forensic and clinical contexts. The present study demonstrated that the skull undergoes accelerated development during the first two years of life, gradually slowing down after the third year. Additionally, the prevalence of brachycephaly decreases with age, consistent with a previous finding^16^. This highlights that the most pronounced changes in cranial proportions occur during early infancy.

Cranial shape and size differ significantly between sexes, with females showing higher CI values, indicating relatively narrower and shorter skulls compared to males in this study. This finding is consistent with Koizumi, Komuro, Hashizume, Yanai^7^, but in contrast with the results of Nam, Han, Eom, Kook, Kim^8^. These differences may reflect population variation and the influence of genetics, environment, and sex hormones on skull development. As skeletal growth is shaped by hormonal, dietary, cultural, and environmental factors, such aspects should be considered when examining sex-based cranial variation^17^.

Several methods have been developed to evaluate head shape variations, including the use of percentile distributions to assess the severity of dolichocephaly and brachycephaly^15^. This classification allows for more accurate diagnosis and follow-up by including a severity index. In this study, the CI value for assessing these conditions was lower than that reported in the Korean population^8^, likely due to differences in sample size and population characteristics. These differences highlight the importance of considering age-specific CI values tailored to each population for more precise assessments and better clinical outcomes.

Variations in CI values have been reported within the Malaysian population. The present study demonstrated higher values (males: 83.91, females: 84.13) compared with one study (males: 81.55, females: 79)^10^, whereas another reported slightly higher values (males: 84.8, females: 85.2)^9^. Differences in CI values between studies may be due to variations in sample size, demographics, measurement methods, and population composition. In addition, earlier studies^9^^,^^10^ focused only on Malay individuals and may not reflect the full diversity of CI values across Malaysia’s multi-ethnic population.

**Fig. 5:**
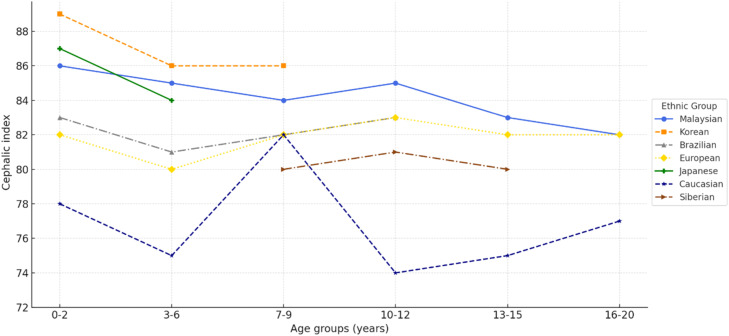
CI comparison between the Malaysian population and other populations.

Variations in CI and head shape among individuals are likely influenced by a combination of genetic, developmental, environmental, and hormonal factors^18^. Comparative studies show that children in Asian countries^7^^,^^8^ have relatively higher CI values than children in the United States^15^, Europe^19^, and Brazil^20^ (Fig. 5). These differences are largely attributed to infant sleep practices, with supine positioning in Asia contributing to the prevalence of brachycephaly^21^. In contrast, prone positioning in the United States was traditionally associated with mesocephaly, although the introduction of the “Back to Sleep” campaign has been linked to a shift toward brachycephaly^2^. The present findings further demonstrate that the prevalence of brachycephaly decreases with age, reflecting a natural adjustment in CI and offering valuable insight into its developmental course^22^ (Fig. 5).

Cranial deformities, such as brachycephaly, may have a profound impact on neurological and psychosocial development. These deformities may contribute to complications such as jaw misalignment, alterations in facial growth, and breathing difficulties, including obstructive sleep apnea^23^^,^^24^. In addition, children with cranial deformities often experience delays in gross motor skills^25^^,^^26^ and may show lower cognitive and academic performance in adolescence, as these conditions can directly impact brain development^26^. Factors like sleeping position, lack of tummy time, weak neck muscles, and low activity levels may also contribute to these developmental delays^25^. Therefore, early diagnosis and treatment may help prevent complications and promote better motor skills, cognitive function, and overall well-being.

Managing brachycephaly involves various treatments such as physiotherapy, repositioning therapy, surgery, osteopathic manipulation, and helmet therapy^27^. A study found that helmet therapy was more effective than repositioning^21^; however, another study suggested no significant difference between helmet therapy and physiotherapy^28^. However, helmet therapy has shown high success rates, with^23^ reporting 81.4 % of patients achieving a final CI < 90 %, while^29^ found a 98.5 % success rate, likely due to higher compliance and longer treatment duration. Therefore, the new CI classification could serve as a valuable tool in guiding these interventions by providing a more precise assessment of cranial shape severity, allowing clinicians to customize treatment strategies for better patient outcomes.

The use of CT imaging in children raises ethical concerns due to radiation risks, as they are more sensitive than adults. Studies show that radiation exposure from CT scans increases the risk of cancers such as leukemia, skin, breast, brain, and thyroid cancers^30^^,^^31^. Therefore, it is crucial to raise awareness among healthcare professionals and apply strict radiological protection measures. This includes following the ALARA (as low as reasonably achievable) principle to minimize radiation while maintaining image quality for accurate diagnosis^32^. Medical practitioners must ensure that CT scans are justified, unnecessary exposure is avoided, and radiation doses are carefully monitored. Ultimately, the benefits of CT imaging must always outweigh the risks, particularly in pediatric cases^30^.

In conclusion, this study demonstrates that CI values are reliable markers of cranial growth and development, useful for monitoring skull shape over time and evaluating abnormal head sizes, with potential links to brain volume and cognitive ability^22^. Malaysian subadults showed higher CI values than most other populations^5^^,^^15^^,^^19^^,^^20^, rendering existing classifications^7^^,^^8^^,^^22^^,^^23^ unsuitable for this population. The proposed Malaysian-specific classification more accurately reflects the country’s multi-ethnic cranial proportions and growth patterns. It provides a valuable reference for detecting, managing, and treating cranial deformities, with important applications in anthropology, anatomy, and forensic medicine.

## Ethics statement

Ethical approval was obtained from the Medical Research Ethics Committee, University Malaya Medical Centre (MREC ID NO: 202147–10039). The requirement for informed consent was waived by the Medical Research Ethics Committee, University Malaya Medical Centre, because this is a retrospective study based on CT images obtained from the hospital archive system. In addition, all procedures conducted in this study involving human participants were in compliance with the guidelines and regulation standards of the national research committee and with the 1964 Helsinki declaration.

## Authors’ contributions

Sharifah Nabilah Syed Mohd Hamdan: Conceptualization; formal analysis; investigation; writing-original draft.

Rabi’ah Al-Adawiyah Rahmat: Conceptualization; investigation; data curation.

Selva Malar Munusamy: Formal analysis.

Norliza Ibrahim: Methodology; Formal analysis; data curation; writing-review & editing.

## Funding

This work received research management fund (project no RMF 0637-2021) from Universiti Malaya.

## Data availability statement

The data supporting the findings of this study can be obtained from the corresponding author upon reasonable request.

## Declaration of competing interest

The authors declare no conflicts of interest.
